# Can an antimicrobial stewardship program reduce length of stay of immune-competent adult patients admitted to hospital with diagnosis of community-acquired pneumonia? Study protocol for pragmatic controlled non-randomized clinical study

**DOI:** 10.1186/s13063-015-0871-2

**Published:** 2015-08-14

**Authors:** Giulio DiDiodato, Leslie McArthur, Joseph Beyene, Marek Smieja, Lehana Thabane

**Affiliations:** Department of Critical Care Medicine, Royal Victoria Regional Health Centre, Barrie, Ontario L4M 6M2 Canada; Pharmacy, Royal Victoria Regional Health Centre, Barrie, Ontario L4M 6M2 Canada; Department of Clinical Epidemiology & Biostatistics, McMaster University, Hamilton, Ontario L8S 4L8 Canada; Department of Pathology and Molecular Medicine, McMaster University, Hamilton, Ontario L8S 4L8 Canada

**Keywords:** antimicrobial stewardship, community-acquired pneumonia, length of stay, natural experiment, pragmatic clinical trial, stepped-wedge design, time-dependent bias

## Abstract

**Background:**

Pneumonia is responsible for a large proportion of hospital admissions and antibiotic utilization. Physician adherence to evidence-based pneumonia management guidelines is poor. Antimicrobial stewardship programs (ASPs) are an effective intervention to mitigate against unwarranted variation from these guidelines. Despite this benefit, ASPs have not been shown to reduce the length of stay of hospitalized patients with pneumonia. In immune-competent adult patients admitted to a hospital ward with a diagnosis of community-acquired pneumonia, does a multi-faceted ASP utilizing prospective chart audit and feedback reduce the length of stay, compared with usual care, without increasing the risk of death or readmission 30 days after discharge from hospital?

**Methods/Design:**

Starting on 1 April 2013, all consecutive immune-competent adult patients (>18 years old) admitted to a hospital ward with a positive febrile respiratory illness screening questionnaire and a diagnosis of pneumonia by the attending physician will be eligible for inclusion in this non-randomized study. All eligible patients who fulfill the ASP review criteria will undergo a prospective chart audit, followed by an ASP recommendation provided to the attending physician. The attending physician is responsible for implementing or rejecting the ASP recommendation. This is a modified stepped-wedge design with a baseline data collection period of 3 months, followed by non-random sequential introduction of the ASP intervention on each of four hospital wards in a single community-based, academic-affiliated 339-bed acute-care hospital in Barrie, ON, Canada. The primary outcome measure is hospital length of stay; secondary outcome measures include days and duration of antibiotic therapy, and inadvertent adverse outcomes of 30 day post-discharge mortality and hospital readmission rates. Differences in outcome measures will be assessed using extended Cox regression analysis. Time to ASP intervention is included as a time-dependent covariate in the final model, to account for time-dependent bias.

**Discussion:**

By designing a pragmatic clinical trial with unique design and analytic features, we not only expect to demonstrate the effectiveness of a real-world ASP, but also provide a model for program evaluation that can be used more broadly to improve patient safety and quality of care.

**Trial registration:**

ClinicalTrials.gov NCT02264756.

## Background

### Community-acquired pneumonia

Community-acquired pneumonia (CAP) is defined as an acute infection of the lower respiratory tract in patients residing outside of hospital for 90 days or longer before presentation [1]. Residents of long-term care homes or nursing homes are frequently diagnosed as having a microbiologically variant form of CAP, but there is little evidence to support this classification system [2, 3]. There is no gold-standard diagnostic test or set of criteria for CAP [4], so the diagnosis is made clinically using a constellation of clinical signs and symptoms and diagnostic tests.

In Ontario, pneumonia is the leading cause of death from bacterial infections and accounts for over 18,000 years of life lost annually owing to premature mortality [5]. Pneumonia accounts for the majority of antibiotic utilization in both hospital and outpatient settings [6, 7]. Evidence-based guidelines for the diagnosis and management of pneumonia are available to physicians [1, 8–12]. Adherence to these evidence-based guidelines is associated with reductions in both mortality and antibiotic utilization [13–18].

### Antimicrobial stewardship

Antimicrobial stewardship is defined as any intervention that minimizes unwarranted variation in antimicrobial utilization from evidence-based best practice with the intent of improving patient safety and quality of care [19]. ‘Unwarranted’ refers to the absence of patient- or disease-specific reasons to justify practice variation from evidence-based practice standards. Antimicrobial stewardship can be operationalized in many different ways, but prospective audit and feedback (persuasive approach) and restricted antimicrobial prescribing policies (restrictive approach) appear to be the most efficacious interventions to achieve the goals of antimicrobial stewardship [19, 20]. Antimicrobial stewardship programs (ASPs) have demonstrated efficacy in improving antimicrobial prescription and reducing rates of hospital-acquired infections [20]. Antimicrobial stewardship programs directed at CAP patients have demonstrated reductions in mortality [15, 21], but have failed to demonstrate reductions in length of stay [20]. Of note, the study by Fine *et al*. [22] used a cluster randomized controlled trial design to examine the impact of an ASP on length of hospital stay in CAP patients. This was the only study to model length of stay as a time-to-event occurrence; differences between the length of stay in intervention and control groups were assessed by survival analysis. The intervention consisted of a prospective chart audit starting on day 3 of hospitalization and physician feedback in the form of a recommendation suggesting the optimal timing of conversion from intravenous to oral antibiotics. The intervention was modeled as a time-invariant dichotomous variable in the final model, despite the fact that the timing of the recommendation varied by up to 7 days from the time of enrollment. The hazard ratio for discharge was 1.16 (95 % confidence interval, 0.97–1.38) for the intervention group, suggesting a non-significant reduction in length of stay of 16 %. It is unknown whether this hazard ratio would have reached statistical significance if the intervention was modeled as a time-dependent covariate in the final model, as unaccounted-for time-dependent bias might have diluted the final intervention effect point estimate [23].

### Research question

In immune-competent adult patients admitted to a hospital ward with a diagnosis of CAP, does a multi-faceted ASP utilizing prospective chart audit and feedback reduce the length of hospital stay and days and duration of antimicrobial therapy, compared with usual care, without increasing the risk of death or readmission 30 days after discharge from hospital?

## Methods/Design

### Setting

All participants will be admitted patients of the Royal Victoria Regional Health Centre, a 339-bed community-based, university-affiliated, acute-care hospital located in Barrie, Ontario, Canada. This centre is the only hospital serving the 128,000 residents of Barrie. Access to acute medical care for Ontario residents, including all hospital services, is publicly funded by the Ministry of Health and Long-Term Care, the provincial agency responsible for funding and oversight of Ontario’s Medicare system. All patients enrolled in the study will be admitted to one of four medical wards. All study patients will be admitted to a hospitalist service. Admission to any medical ward is controlled by bed allocation, a non-medical administrative service within the hospital that is responsible for patient flow and assigning patient care. Hospitalists are not assigned to any one specific medical ward, but provide care across all medical wards.

### Population

Starting 1 April 2013, all consecutive adult patients (≥18 years old) with the following inclusion criteria will be screened for enrollment in the study [5]:

1. Screen positive for febrile respiratory illness on admission to hospital [24], and
2. Diagnosed with pneumonia by the admitting physician (acute exacerbations of chronic obstructive lung disease are considered within the definition of pneumonia for the purposes of this study, as they are commonly treated with the same antimicrobial regimens as patients with pneumonia), and
3. Admitted to a medical ward.

All patients who meet these criteria will be eligible for the intervention except for those with the following exclusion criteria [5]:

1. Hospitalized for ≥48 consecutive hours in the preceding 3 months, or
2. Receiving immunosuppressants (defined as ≥40 mg prednisone daily, or steroid equivalent, for ≥2 weeks preceding hospitalization or any other immunosuppressant used for systemic illness or to prevent transplant rejection), or
3. Neutropenic (defined as a polymorphonuclear count ≤0.5 × 10^9^ cells/l) for any cause, or
4. Immunocompromised (defined as having leukemia, lymphoma, HIV with CD4 cell count ≤200, splenectomy or receiving cytotoxic chemotherapy), or
5. Admitted to high-acuity units, such as intensive care units, or
6. Require mechanical ventilation, either non-invasive or invasive, or
7. Have a life expectancy of ≤3 months (palliative).

### Intervention

All eligible patients with CAP who meet the ASP review criteria will receive the ASP intervention (ASP-i). This intervention consists of a prospective chart audit and physician feedback (persuasive) approach [25]. The ASP members who conduct all the audits and make recommendations consist of a pharmacist trained in infectious diseases (LM) and a physician trained in infectious diseases (GD). All patients are reviewed by both members. The ASP-i recommendations are guided by the Infectious Diseases Society of America CAP guidelines [1] and the Canadian Thoracic Society guidelines for the management of chronic obstructive pulmonary disease [26]. The possible ASP-i recommendations are based on those recommended by the UK National Health Service [25] and include:

1. No change to current care
2. Discontinue antibiotics
3. Intravenous to oral conversion
4. Duration of therapy
5. Dosing change
6. Narrow or broaden spectrum of therapy.

The ASP-i recommendations are not mutually exclusive. All recommendations are documented in the patient’s electronic medical record and communicated directly to the attending physician by the ASP members.

### Study design

This is a pragmatic controlled non-randomized clinical study intended to measure the effectiveness of a ‘real-world’ program [27]. The ASP-i will be implemented in a modified stepped-wedge design [28]. Baseline patient data will be collected for all enrolled patients on each of the medical wards for the first 3 months of the study, and then the ASP-i will be introduced to each medical ward in a non-randomized sequential fashion in 2-month intervals until all medical wards are exposed to the intervention. This design was chosen for several reasons; the preponderance of evidence suggests that antimicrobial stewardship interventions are beneficial to patient safety and quality of care [29]; there are human resource limitations in rolling out the program simultaneously to all wards; and the design has the advantage of a contemporaneous control group for comparison. The unit of randomization in this study could have been the four medical wards; however, randomization of the wards was not included in the design. The allocation of CAP patients to one of the four medical wards is controlled by bed allocation, an administrative branch of the hospital. Bed allocation was blinded to the study and the process of patient allocation is solely dependent on bed availability. In addition, the patients, regardless of their ward location, were all admitted to the hospitalist service. The hospitalist service provides coverage across all four medical wards and has no influence on the allocation of patients to any of the wards. In essence, the patients will be ‘naturally’ allocated to one of the four medical wards and one of the attending hospitalists by a bed allocation process that is blinded to the intervention, so that the additional randomization of the wards themselves should provide very little benefit with respect to minimizing selection bias. In addition, the four medical wards can all accommodate patients with CAP; however, one of the medical wards has historically accommodated more CAP patients than the other three. As a result, this ward was chosen as the first ward to receive the ASP-I, as this would ensure that the maximum number of CAP patients would have earlier access to the ASP-i. The ethical principle of utilitarianism was used to guide the order of ward exposure to the ASP-i. The target of the intervention is the most attending physician (hospitalist) , while the unit of analysis will be individual patient outcomes adjusted for potential clustering effects within hospital wards.

### Outcome measures

The primary outcome measure is length of hospital stay, measured in minutes from the documented time of admission to the documented time of discharge (or censoring). These times are determined and entered into the patient’s electronic medical record by bed allocation staff who are blinded to the intervention. The secondary outcome measures are days and duration of antibiotic therapy. The start and stop dates corresponding to antibiotic administration while the patient is admitted to hospital are entered into the patient’s electronic medical record by pharmacy assistants who are blinded to the intervention. All inpatient antibiotic administration is verified through the paper-based medical administration record by a pharmacy assistant blinded to the intervention. The number of days of antibiotic therapy is defined as the total number of days of all antibiotics administered to the patient both while in hospital and after discharge. The duration of antibiotic therapy is defined as the number of days that the patient received antibiotics both while in hospital and after discharge. For example, if a patient received two antibiotics for 4 days over the same time period, then the days of antibiotic therapy is 8 days and the duration of antibiotic therapy is 4 days. The relevance of collecting both days and duration of therapy is that days of therapy is considered a valid metric for monitoring and comparing antibiotic utilization both within and across hospitals, whereas duration of therapy is necessary for determining adherence with best-practice treatment guidelines [30]. All antibiotic data from discharged patients will be extracted (by LM or GD) from the physician discharge summary and all patients will be contacted 30 days after discharge to verify their adherence with the prescribed antibiotic record from the discharge summary. Other secondary outcome measures include inadvertent adverse outcomes of readmission and mortality 30 days after discharge from hospital. Readmission to hospital is determined through telephone survey with the patient and verification through the Royal Victoria Regional Health Centre patient database. Survival is determined through telephone survey with the patient. The status of patients who cannot be reached by telephone will be verified through the Ontario vital statistics registry.

### Participant timeline

Enrollment of patients started 1 April 2013. The study is expected to enroll patients until 31 March 2015. All consecutive patients who meet the inclusion criteria and have no exclusion criteria will be eligible for the intervention. The ASP-i intervention may be implemented any time from 48 hours after admission in those patients who meet the criteria for ASP review. All patients who have not experienced an outcome measure at 14 days after admission will be censored from the study. Patients who die or are transferred from the ward (to the intensive care unit or other hospital) will also be censored. Patients who are discharged from hospital and are not censored will be contacted 30 days after discharge to determine their adherence with antibiotic prescription (if relevant), survival status, and readmission status.

### Sample size

The sample size expected for the current study is ‘fixed’ and has been previously estimated at between 400 and 500 CAP patients per calendar year [31]. The accrual period will be 24 months. Assuming that 70 % of patients in the control arm will achieve the primary outcome of being discharged alive from hospital, and setting power = 0.8 and statistical significance (two-sided) α = 0.05, the detectable ASP-i effect size is estimated to be up to an approximately 20 % reduction in length of stay [32]. The *stpower cox* command in STATA/MP 13.1 for Mac will be used for the calculation. 

### Recruitment

All consecutive immune-competent adult patients admitted to a hospital ward with a diagnosis of CAP will be enrolled in the study, to ensure maximum enrollment.

### Assignment of intervention

Given the ‘naturally’ blinded allocation process of patient admission to one of four medical wards, the pressure to demonstrate an early impact to administrators and the ethical principle of utilitarianism, the ASP-i will first be implemented in the medical ward most likely to accommodate the majority of CAP patients. The remainder of the wards will be sequentially introduced to the ASP-I in order from the ward most likely to care for the most to that most likely to care for the least number of CAP patients, based on historical admission patterns.

### Blinding

It is not possible to blind the ASP members or the attending physicians to the ASP-i. The ASP members are not blinded to the outcome assessment, but this should have minimal risk of bias, given the objective nature of the primary and secondary outcome measures. The principal investigator is also responsible for data analysis, given the absence of biostatistical expertise at the hospital, the absence of funding to support external biostatistical services, and the need to create quarterly reports for the hospital administrators, as required by the employment contract between the Royal Victoria Regional Health Centre and the principal investigator.

### Data collection

All patient-level data will be extracted from the patients’ electronic medical records by the ASP members, using a standardized electronic data collection form that is accessible on a portable tablet computer. The elements of the data dictionary defining the variables and instructions for data abstraction have been preprogrammed into each variable’s respective field and can be accessed at the point of care by touching the field name. Other techniques to ensure internal validity of the data include preprogrammed data integrity constraints, such as range checking and logical data edits and predefined value lists in drop-down menus for the vast majority of data elements. Summary descriptive statistics for all continuous variables will be calculated within each patient record to permit real-time review, to identify any potential outlying values. The external validity of the data will be assessed by an external reviewer on a biannual basis using a random sample of 10 % of the database. The data elements that will be audited by the external reviewer include only those elements that are included as either outcomes or covariates in the final statistical model and are feasible for validation against an objective source. Access to patient-level data is restricted to ASP members. All patient-level data will be protected according to the Personal Health Information Protection Act of Ontario regulations [33].

### Statistical methods

An extended Cox regression analysis that models the ASP-i as a time-variant covariate will be used to compare the primary and secondary outcome measures between the control and intervention groups [34]. Violations of the proportional hazards assumption for each covariate will be identified using the method of Schoenfeld residuals combined with the graphical method of log-log survival curve analysis [34]. Results will be reported as hazard ratios with 95 % confidence intervals. Patients who remain in hospital beyond 14 days will be administratively censored. Competing events, such as death or transfer from a medical ward to a critical care unit will be assumed to be non-informative conditional on the covariates included in the final model [34]. Time to ASP-i will be modeled as a time-variant covariate in the final model to account for any time-dependent bias [23]. The ASP-i exposure will be coded as a dichotomous variable; 0 = no ASP-i, switching to 1 = ASP-i at the time of the ASP-i and remaining 1 throughout the remainder of the follow-up time. This method treats a patient who has received an ASP-i as a non-ASP-i patient prior to the intervention. Other variables known to be associated with the primary and secondary outcome measures will also be included in the final model [5], and include; age, sex, Charlson comorbidity index, CURB-65 score, time (days) to clinical resolution, and complications from pneumonia, such as empyema. Fixed effects of wards on the outcomes will accounted for by including them as indicator variables in the final model. Maturation of ASP-i effect on outcomes over time will be adjusted by including a categorical time variable in the final model (time variable will be defined as ‘quarter’ from start of study). A dichotomous variable for acceptance or rejection of the ASP-i will be part of an interaction term with the control/intervention group variable to permit a per-protocol analysis. Sensitivity analysis will be done using a competing events Cox regression model to determine the impact of the assumption of the non-informative nature of events, such as death and admission to an intensive care unit [35].

### Monitoring

The hospital administration requires quarterly reporting of primary and secondary outcome variables. Secondary adverse outcome measures such as mortality and readmission rates to hospital within 30 days after discharge will be monitored in real-time by preprogrammed calculation of relative risk ratios adjusted by the LACE score [36]. Relative risk ratios >1.2 for death or readmission at 30 days post-hospital discharge for the intervention group will be used to notify the hospital administration that an inadvertent adverse event might be due to the intervention, and an external safety monitoring committee of the Hospital Pharmacy and Therapeutics committee will be responsible for auditing the program. This relative risk ratio was chosen by the hospital administration as a reporting trigger.

### Research ethics

The study has received approval from the research ethics board of the Royal Victoria Regional Health Centre (in an unnumbered research ethics board document entitled ‘*Intervention study of the impact of an antimicrobial stewardship program (ASP) on the length of stay of patients admitted to Royal Victoria Regional Health Centre with a diagnosis of community-acquired pneumonia (CAP) using a before-and-after quasi-experimental study with a control group’.*) A waiver of informed consent was approved by the research ethics board because of the nature of the pragmatic clinical trial [37, 38], based on the minimal risk of harm to patients and the fact that any ASP recommendation that is implemented will have required that the attending physician receive informed consent from the patient as per the usual process of care. A waiver for informed consent was also granted to the ASP members to contact the patients by phone 30 days after discharge, in order to ensure the safety of the program with respect to its impact on readmission and mortality rates. Any protocol amendments or violations will be reported to the research ethics board for review.

## Discussion

Pragmatic clinical trials are designed to embed research into practice, to reduce the delay observed in translating clinical research into practice [39]. Pragmatic clinical trials not only produce results that are immediately relevant to patients and stakeholders, but these results emerge from methodologically reliable designs [40]. Despite these qualities, pragmatic clinical trials made up only 2 % of registered clinical trials in ClinicalTrials.gov in the calendar year 2013 (search terms: ‘pragmatic* or practical* or comparative-effective*’ versus ‘clinical trials’ (accessed July 6, 2014)). In Canada, peer-reviewed research funding agencies dispensed over 45,500 grants and awards totalling more than $12 billion from 1999/2000 to 2013/2014, but only 480 of these totalling $118 million were used to support pragmatic clinical trials (Canadian Research Information System, http://webapps.cihr-irsc.gc.ca/cris/search; search terms: ‘pragmatic, practical, comparative-effectiveness’ (accessed July 6, 2014)). None of these grants and awards were used to support community-based pragmatic clinical trials. While this trial is designed to demonstrate the effectiveness of an ASP-i designed to reduce the length of hospital stay of CAP patients, an important finding that has yet to be published, it is also meant to demonstrate both the feasibility and value of research that is conceived and conducted by community-based healthcare providers. Given that more than 60 % of all inpatients in Ontario are admitted to community-based hospitals [41], it is essential that community-based healthcare providers are invited to suggest locally relevant research studies that are eligible for dedicated peer-reviewed funding opportunities. This study is intended to be an example and guide for those community-based healthcare providers interested in contributing to improving their healthcare programs and systems by using pragmatic clinical trial designs.

There are no previous studies examining the effectiveness of a prospective audit and feedback ASP-i on important patient outcomes that account for time-dependent bias. In general, these ASP-interventions are time-dependent interventions that have been incorrectly included as time-invariant covariates in statistical models. From an epidemiological perspective, these ASP-interventions are not present at the time of hospital admission but are ‘acquired’ at some time after admission to hospital. In the biased analysis, patients who are exposed to an ASP-i are analyzed as though the ASP-i had occurred at the time of hospital admission, thereby increasing the denominator of the length-of-stay hazard. The result of this mis-specification is a reduction in the length-of-stay hazard in the ASP-i group. The resultant length-of-stay hazard ratio will be biased and closer to 1, suggesting the absence of any beneficial ASP-i effect [23]. This study is the first to account for time-dependent bias by including ASP-i as a time-variant covariate in the final statistical model.

This study has several challenges to its internal and external validity. First, the absence of medical ward randomization could introduce selection bias. However, the allocation of patients to the ward and their medical provider is determined through a ‘natural’ allocation process that is blinded to the study, thus minimizing this potential source of bias. Secondly, the ASP members are not blinded to the intervention, outcome measure assessment, or data analysis. However, the likelihood of introducing bias is minimal because the primary and secondary outcome measures are stringently defined and objective. Thirdly, the risk of contamination is significant given that the hospitalists provide coverage across the four medical wards. It is conceivable that their behaviour will change over time as they become conditioned to the ASP-i and that this behaviour change might then precede the ASP-i as the study evolves over the 2 year period. The consequence of this contamination would be to bias the outcome hazard ratios toward a value of 1 or no ASP-i effect. However, a recent Cochrane review of the impact of audit and feedback on physician behaviour demonstrated only a 4.3 % (interquartile range, 0.5 % to 16 %) absolute increase in adherence with best practice [42], suggesting that the risk of contamination in this study might be small given the inherent resistance of physicians to changes in behaviour. In addition, the rate of ASP-i consults per month (defined as the ratio of the number of ASP-i consults to the number of eligible patients) will be modeled as time series data, and an assessment using regression analysis and the Durbin alternative test will be used to detect any serial correlation between the previous months’ ASP-i and the subsequent rate of ASP-i consults. Evidence of serial correlation between preceding ASP-i and subsequent rates of ASP-i consults would suggest possible contamination. Of course, contamination in this study might simply represent that the ASP was able to effectively change physician behaviour in a positive manner; a desirable impact for patient care, albeit with some undesirable effects on exposure-outcome evaluation. Fourth, the timing of the ASP-i at ≥48 hours after admission restricts the option of recommending earlier conversions from intravenous to oral antibiotics in eligible patients. Previous studies have suggested that earlier conversions might reduce length of stay [31, 43]. If the causal mechanism for reducing length of stay is through this earlier conversion, then this study might not be able to detect this ASP-i effect. As a single hospital site study, the applicability of the results to other hospitals and programs is unknown. However, a multi-site study using the same design, intervention, and analysis is currently being planned. Submissions of the proposal have been made to each participating hospital’s research ethics board. The anticipated start date of this multi-site study is 1 May 2015.

We believe that the ASP-i will lead to shorter lengths of hospital stay for CAP patients and reduced administration of unnecessary antibiotics, both of which should result in reduced rates of hospital-acquired complications and reduced healthcare costs. In addition, this study offers a model for community-based healthcare providers to design and conduct a pragmatic clinical trial for the purpose of programmatic evaluation. By involving community-based hospitalized patients and their healthcare providers in translating research into practice, access to improvements in our healthcare system will finally be more equitably distributed and shared.

## Trial status

The study has enrolled 582 patients. The study will continue to enroll patients until 31 March 2015.

## Abbreviations

ASP: Antimicrobial Stewardship Program
ASP-i: Antimicrobial Stewardship Program intervention
CAP: community-acquired pneumonia
